# Physical and Physiological Characteristics of Elite Male Handball Players from Teams with a Different Ranking

**DOI:** 10.2478/hukin-2013-0051

**Published:** 2013-10-08

**Authors:** Pantelis T. Nikolaidis, Jørgen Ingebrigtsen

**Affiliations:** 1 Department of Sports Medicine and Exercise Biology, Faculty of Physical Education and Sport, Athens, Greece.; 2 Department of Sports, Centre for Practical Knowledge, University of Nordland, Bodø, Norway.

**Keywords:** physical fitness, anthropometry, anaerobic power, discriminant variables, sport excellence, handball

## Abstract

The aim of this study was to examine possible discriminant physical and physiological characteristics between elite male handball players from elite teams with different league rankings. Players from three teams (A, B and C), which competed in the first league of the Greek championship during the season 2011–2012 participated in the study. Team A finished first, B came second and C came eighth out of eleven clubs. Teams A and B also participated in European Cups, and team A won the European Challenge Cup. The players (n=44) were examined for anthropometric characteristics and performed a series of physical fitness tests. Players from teams A and B were taller (6.2 cm (0.7;11.7), mean difference (95% CI) and 9.2 cm (4.0;14.5), respectively), and had a higher amount of fat free mass (6.4 kg (1.1;11.8) and 5.4 kg (0.2;10.5)) compared to those of team C. Players from team A performed better than players from team C in the squat jump (5.5 cm (1.0;10.0)), the countermovement jump without (5.5 cm (0.4;10.6)) and with arm-swing (6.0 cm (0.7;11.3)) and in the 30 s Bosco test (5.7 W·kg−1 (1.2;10.2)). Also, players from team A outperformed team B in mean power during the Wingate anaerobic test (WAnT, 0.5 W·kg−1(0;0.9)) and in the Bosco test (7.8 W·kg−1 (3.4;12.2)). Overall, players from the best ranked team performed better than the lowest ranked team on WAnT, vertical jumps and the Bosco test. Stepwise discriminant analysis showed that stature and mean power during the Bosco test were the most important characteristics in TH players, accounting for 54.6% of the variance in team ranking. These findings indicate the contribution of particular physical fitness components (stature, fat free mass and anaerobic power) to excellence in TH. In addition, the use of the Bosco test as an assessment tool in talent identification and physical fitness monitoring in this sport is further recommended.

## Introduction

Handball is played by approximately 19 million players distributed between 800 thousand teams across 167 member federations listed by the International Handball Federation. The sport requires that handball players possess various technical skills (e.g. shooting and passing) and fitness components (e.g. jumping ability, speed, endurance) to reach the highest levels (Marques and Gonzalez-Badillo, 2006; Ronglan et al., 2006; Buchheit et al., 2009; Ingebrigtsen and Jeffreys, 2012; Ingebrigtsen et al., 2012). Therefore, the investigation of key factors and characteristics that can distinguish between high- and low-class players is continually ongoing among practitioners and scientists (Granados et al., 2007; Buchheit et al., 2009).

The available research on handball players indicates that physiological characteristics such as speed, strength and jumping abilities are dependent on the level of competition (Lidor et al., 2005; Gorostiaga et al., 2006; Marques and Gonzalez-Badillo, 2006; Ziv and Lidor, 2009). This suggests that testing of physiological characteristics could be used for the detection of successful players. Since most of the previous studies have been conducted on non-elite players, further investigation into the characteristics of elite players is needed. Such data would help handball players to evaluate their physical characteristics and physiological abilities, and to design specific training programs (Ingebrigtsen et al., 2012).

Furthermore, previous research has indicated that certain physical characteristics are related to high-level handball performance (Gorostiaga et al., 2005; Lidor et al., 2005; Ziv and Lidor, 2009). In particular, a high body mass and stature is commonplace among players (Ziv and Lidor, 2009), and evidence suggests that international junior and senior players have increased their body mass and stature in recent years (Ingebrigtsen et al., 2012). These findings also indicate relatively heterogeneous physical characteristics across all player positions in the team (Lidor et al., 2005; Ziv and Lidor, 2009), although some studies have reported that wings were lighter and smaller when compared to players in other positions (Vila et al., 2011). However, conducting larger studies on physical (e.g. body fat percentage, fat free mass (FFM) and somatotype) and physiological (e.g. working capacity at heart rate 170 beats • min−1 (PWC170), Wingate anaerobic test (WAnT) and 30 s Bosco test) characteristics that have previously not been extensively investigated could enhance our understanding of the elite handball player.

Hence, to increase our knowledge of elite performance in male handball, and to collect up-to-date data related to high-level male players, more investigations need to be undertaken. This knowledge could be used by coaches in order to make better selection of players and to design training programs according to the specific needs of each player. Therefore, the primary purpose of the study was to examine the possible discriminant physical and physiological characteristics between elite male handball players from teams with different league rankings. We hypothesized that players from the higher ranked teams would have superior physical and physiological characteristics.

## Material and Methods

### Participants

All players (n = 44) volunteered for this study. Informed consent was received from all participants or their guardians, in case of underaged players (age < 18 yr, n = 5), after verbal explanation of the experimental design and potential risks of study. Exclusion criteria included history of any chronic medical conditions and use of any medication. All participants visited the laboratory once and underwent a series of anthropometric and physiological measures. The study was performed in accordance with the Declaration of Helsinki and approved by the local Institutional Review Board.

*Design.* In this investigation, a cross-sectional, descriptive-correlation design was used to examine the relationship between physical and physiological characteristics, and sport performance in handball. Players from three teams, A, B and C, which competed in the first league of the Greek championship during the 2011–2012 season, participated in this study. Team A finished first, B came second and C came eighth out of eleven clubs. Teams A and B also participated in European Cups, and team A won the European Challenge Cup. Testing procedures were performed during the competitive period of the season. Since the teams took part in many competitions (Championship, National Cup and European Cups), it was not possible to devote more days to testing and, therefore, all procedures were carried out in a single day.

### Procedures

Physical measurements included stature, body mass and skinfolds. BMI was calculated as the quotient of body mass (kg) to height squared (m^2^). Body fat (BF) was estimated from the sum of 10 skinfolds (cheek, wattle, chest I, triceps, subscapular, abdominal, chest II, suprailiac, thigh and calf; BF = −41.32 + 12.59 · log_e_x, where × is the sum of 10 skinfolds) (Parizkova, 1978). An electronic weight scale (HD-351 Tanita, Illinois, USA) was employed for body mass measurement (to the nearest 0.1 kg), a portable stadiometer (SECA, Leicester, UK) for stature (0.1 cm), a caliper (Harpenden, West Sussex, UK) for skinfolds (0.5 mm), a small anthropometer (Lafayette Instrument Company, Lafayette, Indiana, USA) for breadths (0.1 cm) and a tape (SECA, Leicester, UK) for circumferences (0.1 cm). The waist-to-hip ratio was calculated as waist circumference divided by hip circumference. A two-component model of body composition divided the body into fat mass (FM), calculated as the product of body mass by BF, and FFM, estimated as the difference between body mass and FM. The anthropometric Heath-Carter method for somatotyping was used to quantify the shape and composition of human body, expressed in a three-number rating representing endomorphy (relative fatness), mesomorphy (relative musculoskeletal robustness) and ectomorphy (relative linearity or slenderness) (Heath and Carter, 1967). All participants performed the following physical fitness tests in respective order:

(a) Sit-and-reach test (SAR). The SAR protocol (Wells and Dillon, 1952) was employed for the assessment of lower back and hamstring flexibility.
(b) Physical working capacity at heart rate of 170 beats·min^−1^ (PWC170). PWC170 was performed according to Eurofit guidelines (Adam et al., 1988) on a cycle ergometer (828 Ergomedic, Monark, Sweden). Seat height was adjusted to each participant, and toe clips with straps were used to prevent the feet from slipping off the pedals. Participants were instructed to pedal with a cadence of 80 revolutions per minute, which was regulated by both visual (ergometer’s screen showing pedaling cadence) and audio means (metronome set at 80 beats per minute). The PWC170 test consisted of three stages, each lasting 3 min, against incremental braking force in order to elicit heart rate between 120 and 170 beats·min^−1^. Based on the linear relationship between heart rate and power output (Bland et al., 2012), PWC170 was calculated as the power corresponding to heart rate of 170 beats·min^−1^ and expressed as W·kg^−1^.
(c) Squat jump (SJ), countermovement jump without (CMJ) and with arm-swing (CMJarm). The participants performed two trials for each jumping exercise and the best result was recorded (Aragon-Vargas, 2000). Height of each jump was estimated using the Opto-jump (Microgate Engineering, Bolzano, Italy), and was expressed in cm.
(d) Bosco test. This test was conducted on the same equipment as the abovementioned jump tests. The participants were instructed to jump as high as possible, while trying to retain short ground contact times (Sands et al., 2004). They were also requested to keep their hands on their waist throughout the test. The mean power during the 30 s test was recorded in W·kg^−1^.
(e) Handgrip strength test (HST). The participants were asked to stand with their elbow bent at approximately 90° and instructed to squeeze the handle of the handgrip dynamometer (Takei, Tokyo, Japan) as hard as possible for 5 seconds (Adam et al., 1988). HST was calculated as the sum of the best efforts for each hand divided by body mass and expressed as kg·kg^−1^ of body mass.
(f) Force-velocity test (F-v). The F-v test was employed to assess maximal anaerobic power (Pmax expressed as W·kg^−1^). This test employed various braking forces that elicit different pedaling velocities in order to derive Pmax (Vandewalle et al., 1985). The participants performed four sprints, each one lasting 7 s, against incremental braking force (2, 3, 4 and 5 kg) on a cycle ergometer (Ergomedics 874, Monark, Sweden), interspersed by 5 min recovery periods.
(g) The WAnT (Bar-Or and Skinner, 1996) was performed in the same ergometer as the F-v did. Briefly, participants were asked to pedal as fast as possible for 30 s against a braking force that was determined by the product of body mass in kg by 0.075. Mean power (Pmean) was calculated as the average power during the 30 s period and was expressed as W·kg^−1^.

### Statistical analysis

Statistical analyses were performed using IBM SPSS v.20.0 (SPSS, Chicago, USA). Data were expressed as mean and standard deviations of the mean (*SD*). One-way analysis of variance (ANOVA), with a sub-sequent Tukey post-hoc test (if difference between the groups was revealed) were used to examine differences in physical and physiological characteristics among the three handball teams. The level of significance was set at α=0.05, and mean difference ± SD together with 95% confidence intervals (CI) was calculated when the post-hoc was necessary. In addition, stepwise discriminant analysis was used for physical and physiological characteristics with team ranking as the dependent variable.

## Results

The ANOVA analysis revealed (Table 1) significant differences between the players of the three teams in stature and FFM. Players from team C had lower stature compared to players from team A (−6.2±2.2 cm (−11.7; −0.7), mean difference±SD (95% CI)) and team B (−9.2±2.2 cm (−14.5; −4.0), respectively). Also, players from team C had lower amounts of FFM compared to the other two teams, with −6.4±2.2 kg (−11.8;−1.1) and −5.4±2.1 (−10.5; −0.2) relative to A and B, respectively.

As shown in Table 2 there were significant between group differences in Pmean, SJ, CMJ, CMJarm and the 30 s Bosco test. The Tukey post-hoc analysis revealed that players in team A scored higher on Pmean than both players in teams B (+0.48±0.18 W·kg^−1^ (0.05;0.92)) and C (+0.46±0.19 W·kg^−1^ (0.01;0.92)), respectively. Players from team A jumped higher than their team C counterparts in SJ (5.5±1.8 cm (1.11;9.9)), CMJ (5.5±2.0 cm (0.5;10.4)) and CMJarm (6.0±2.1 cm (0.8;11.2)), and performed better than players in team B (+7.8±1.8 W·kg^−1^ (3.5;12.1)) and C (+5.7±1.8 W·kg^−1^ (1.4;10.1)) on the 30 s Bosco test.

Stepwise discriminant analysis showed that stature (m) and mean power (W·kg^−1^) during the Bosco test were the most important characteristics in elite handball players (Table 3). These two parameters accounted for 54.6% of the variance in the team performance level.

## Discussion

A main and novel finding in the present study was that players from the best male handball team were found to produce higher mean power output relative to body weight (W·kg^−1^) during the 30 s Bosco jumping test and the WAnT, compared to players from lower ranked teams from the same country. Such differences have not previously been shown. Additionally, the same players jumped higher in the three vertical jump tests, and were both taller and had higher amounts of FFM, compared to their lower ranked counterparts.

### Physiological characteristics

A novel finding of the present study was that players in lower ranked teams produced lower (−5.8 to −7.8 W·kg^−1^) mean power output during a 30 s modified Bosco test compared to players in the best team in the group. Moreover, the average score of all participants (34.3 W·kg^−1^) was superior than values reported by previous studies using the 30 s Bosco test (e.g, in 18–24 yr 18.3 W·kg^−1^ (Fabian et al., 2001); in university athletes 21.3 W·kg^−1^ (Sands et al., 2004), and in volleyball players Bosco 24.8 W·kg^−1^ (Bosco et al., 1982)). To the best of our knowledge, only one study previously reported mean power output in handball players, where a 15 s modified Bosco test was applied (∼26 W·kg^−1^ in Italian national team) (Bonifazi et al., 2001). Therefore, we interpret the present identification of mean power output during continuous vertical jumping as a parameter that discriminates between players according to their team level. In addition, we suggest further use of continuous jumping tests as an integral part of a handball specific test battery when attempting to identify and select future talented handball players.

The present study also revealed that relative mean power output (W·kg^−1^) in the WAnT differentiated between players from the best team and players from the two lower ranked teams. Although a similar study has not been conducted previously in adult players, Bencke et al. (2002) compared mean power in WAnT between elite and non-elite players aged 12.0–12.5 yr, showing higher scores in the former group (8.0 vs. 7.3 W·kg^−1^). To the best of our knowledge, only two studies (Kalinski et al., 2002; Norkowski, 2002) reported mean power output in the WAnT in adult players previously. Kalinski et al. (2002) reported similar values in 76 national level handball players when compared to the present study findings (8.9 vs. 8.3 to 8.8 W·kg^−1^). In addition, these authors showed that handball players had the highest score among basketball, volleyball, rugby and soccer players. Norkowski (2002) also reported similar values (8.8 W·kg^−1^) for 30 national level handball players. Therefore, the ability to maintain maximal effort for repeated actions that last several seconds is important for sport selection, as well as for performance among elite players.

**Figure 1:**
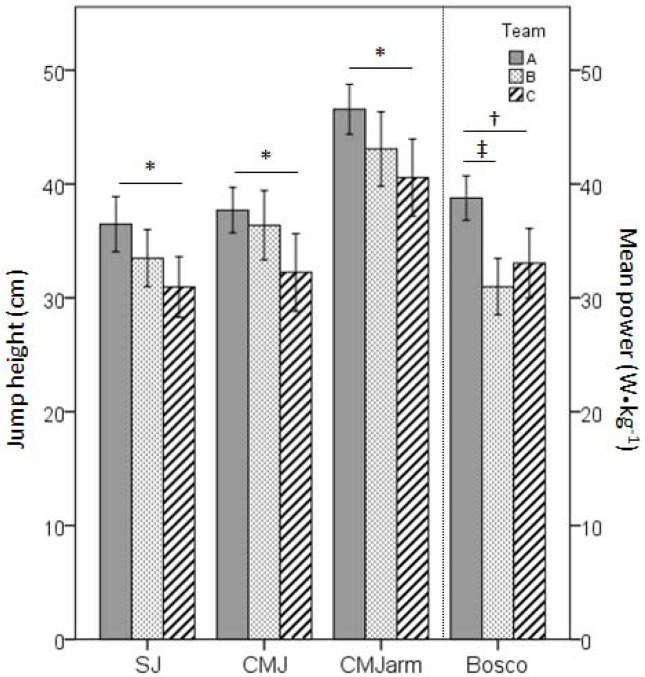
Jumping performances of participants according to their team. SJ=squat jump, CMJ=countermovement jump without arm-swing, CMJarm=countermovement jump with arm-swing and Bosco=mean power during 30 s modified Bosco test. *p<0.05, †p<0.01 and ‡p<0.001 significance level of the Tukey post-hoc test

Furthermore, the current study found that players in the best team jumped substantially higher compared to the lowest ranked team in all jump tests (SJ 5.5 cm, p<0.05, CMJ 5.5 cm, p<0.05, CMJarm 6.0 cm, p<0.05). This was in agreement with the aforementioned study of Bencke et al. (2002) on children, which also revealed higher scores in SJ in elite than in non-elite players (28.5 vs. 23.0 cm). We also examined our findings with regard to previous studies on elite male handball players (Bonifazi et al., 2001; Marques and Gonzalez-Badillo, 2006; Ronglan et al., 2006; Buchheit et al., 2010) Bonifazi et al. (2001) found CMJ performance in Italian national handball players to be 49 cm. This is substantially higher compared to the CMJ performances of all present teams when jumping without an arm swing, and also 6 to 7.5 cm higher compared to the second and third ranked teams in the present group, but only 2.5 cm higher than the players from the best team in this study, when jumping with an arm swing. However, Bonifazi et al. (2001) did not specify whether the athletes used an arm swing during the CMJ. Compared to other previously published CMJ results, our study found that the lower ranked teams had very similar results (43 and 41 vs. 42 cm) in Spanish high level handball players (Marques and Gonzalez-Badillo, 2006), while the higher ranked team had comparable results (47 vs 47 cm) with national handball players (Buchheit et al., 2009). However, the comparison between present and previous jumping ability data is problematic, as it has been shown that the applied measuring equipment plays a significant role on the outcome, and can vary substantially between models (Linthorne, 2001; Glatthorn et al., 2011). Nevertheless, we found the present results to indicate that high vertical jumping ability is beneficial for reaching the elite handball level for male players. Hence, measuring this ability could play a key role when searching for future elite male handball players. Also, we found the present results to indicate that jumping ability could be used to distinguish between players in higher and lower ranked handball teams (Figure 1). Furthermore, when observing Table 2, it is interesting to note that team A had lower standard deviations of the mean than the other teams, i.e., variation in physiological characteristics was smaller in the former team. Therefore, in addition to their superior physiological characteristics, the homogeneity in these characteristics may also discriminate international level players from their national level counterparts.

Considering the intermittent nature of handball, it has been stated that performance is associated with the ability to produce high power in short time periods (anaerobic power) and the ability to recover between such high-intensity activities (aerobic power) (Povoas et al., 2012). While the comparison of the three teams did not reveal significant differences in aerobic power, the differences in the jump tests and the WAnT highlighted the importance of anaerobic power. The better scores of the highest ranked team in the WAnT and Bosco test indicated that the players of this team exhibited higher anaerobic power. Since the WAnT and Bosco test lasted 30 s demanding maximal effort, the mean power produced during these tests was considered to utilize the same energy transfer system. From a physiological point of view, both tests measured the same parameter (anaerobic power), but they used two different motion modes (cycling and jumping, respectively). Comparing these tests, the higher values of power recorded in jumping might be partially attributed to the fact that jumping performance included the stretch-shortening cycle and the myotatic stretch reflex (Fabian et al., 2001). Although both tests had the ability to reveal the same differences among groups (higher values in team A than in teams B and C), the discriminant analysis showed only the ability of the Bosco test to discriminate between teams according to the ranking. Taking into account the significant correlation between the Bosco test and the percentage of muscle fibers type II (Bosco et al., 1983), a high performance in this test might have implications not only on players’ ability to repeatedly perform activities involving jumping, but also on their ability to accelerate and develop maximum speed.

### Physical characteristics

Although the present study strengthen previous indications of high stature being an advantage for handball players (Gorostiaga et al., 2005; Lidor et al., 2005; Ziv and Lidor, 2009; Ingebrigtsen et al., 2012), this is the first study to show that stature can also be used to discriminate between levels of play within the elite population. Moreover, in a review of literature, Ziv and Lidor (2009) showed that stature in male handball players ranged from 174 cm to 189 cm. The latter was reported among the best Spanish players (Gorostiaga et al., 2005), and is comparable to the best teams, but somewhat higher than the lowest ranked team, in the present group. Previous research (Mohamed et al., 2009) has identified stature and sprint shuttle-run ability as traits that discriminate between players according to their level (elite vs. non-elite).

Furthermore, the amount of FFM also significantly distinguished the players of the higher ranked team and the players in the lower ranked team. A high body mass, and especially a high degree of FFM has previously been speculated to be beneficial in handball (Ziv and Lidor, 2009), and therefore we interpret the present findings to strengthen this assumption. However, the subjects had lower absolute amounts of FFM compared to what has previously been shown among players in the best Spanish team (Gorostiaga et al., 2005). Despite the aforementioned difference with regard to FFM, the corresponding difference in BF was not statistically significant. Regarding somatotype, endomorphy scores were in accordance with BF, i.e. lowest, but not statistically significant, scores in the highest rank team. Hence, we speculate that FFM, as well as stature, should be considered when identifying and selecting high-level elite handball players.

Descriptive analyses, and investigations of differences between players in teams with different rankings, are limited to gaining information on these players only and identifying differences between them rather than answering questions related to the cause and effect. The reader should keep this in mind when reading the practical applications given below (i.e. based on the multifaceted nature of handball it is not given that increased jumping ability alone would make a better player). Nevertheless, the present data could serve as reference data for other elite team fitness coaches and players to compare against, and for coaches to take into account when scouting for future elite players. Due to the differences in physiological and physical characteristics among male elite players, and the fact that testing of certain characteristics (i.e. mean power output in the WAnT and 30 s Bosco jumping test, and stature and amount of FFM) might be helpful for selecting future, and discriminating between already elite players, it is recommended that such tests are used when evaluating the level of elite players.

### Limitations

Due to the cross-sectional design of this study, it is not possible to establish whether the physiological characteristics can be trained to improve at such high level competition; for instance, it remains unknown whether a player with lower values of jumping performance has the potential to reach high values in the future. In addition to the physiological characteristics examined, there might have been other parameters (psychological, social, nutritional, technique) that also influence performance and account for parts of the variance in the ranking. For instance, it is reasonable to assume that higher ranked teams employ more professional players than lower ranked teams.

Although we identified two main characteristics (stature and ability to perform continuous jumping) that discriminate players according to their team ranking, these findings should be applied with caution in other populations (e.g. young players).

## Conclusions

For the first time, mean power output (W·kg^−1^) in the 30 s Bosco test and WAnT, were shown to discriminate between players from higher ranked and lower ranked male elite handball teams. Also, vertical jump performances were better among the players on the best team. Furthermore, higher stature and amount of FFM were found in players from the higher ranked teams. This could indicate that both physiological and physical characteristics can be useful for discriminating between elite male handball players.

## Figures and Tables

**Table 1:** Physical characteristics of participants with ANOVA and Tukey post –hoc indicating mean differences between the players of the teams

	Team A (n = 14)	Team C (n = 13)	Team C (n = 13)	ANOVA
Age (yr)	24.0±5.7	27.2±6.7	25.0±5.8	F_2,41_ = 1.16, p = 0.323
Body mass (kg)	87.6±9.0	87.5±9.8	81.8±8.7	F_2,41_ = 1.76, p = 0.185
Stature (cm)	185.1±6.5^C^	188.2±6.1^C^	179.0±4.7^A,B^	F_2,41_ = 9.22, p = 0.000
BMI (kg·m^−2^)	25.6±2.4	24.7±2.4	25.6±2.7	F_2,41_ = 0.60, p = 0.552
BF (%)	16.6±3.6	17.8±4.0	18.6±4.0	F_2,41_ = 0.96, p = 0.392
FFM (kg)	72.8±5.3^C^	71.7±6.2^C^	66.4±5.5^A,B^	F_2,41_ = 4.94, p = 0.012
WHR	0.82±0.03	0.80±0.05	0.81±0.03	F_2,41_ = 0.60, p = 0.553
Endomorphy	3.3±1.0	3.6±1.0	4.1±1.4	F_2,41_ = 1.98, p = 0.151
Mesomorphy	5.2±1.2	4.9±1.1	5.7±1.2	F_2,41_ = 1.54, p = 0.226
Ectomorphy	2.1±1.0	2.5±1.1	1.7±1.0	F_2,41_ = 2.22, p = 0.121

Data are mean±SD. BMI was body mass index, BF body fat percentage, FFM fat-free mass, WHR waist-to-hip ratio.

The letters A, B or C when presenting as exponents denote that the column’s group differs from the respective group

**Table 2:** Physiological characteristics of, and differences between, participants assessed by one-way ANOVA and a Tukey post-hoc

	Team A (n = 14)	Team B (n = 17)	Team C (n = 13)	ANOVA
PWC170 (W·kg^−1^)	3.3±0.5	2.9±0.6	2.9±0.8	F2,39 = 1.89, p = 0.164
Pmax (W·kg^−1^)	13.2±2.3	14.8±1.8	13.9±2.7	F2,38 = 2.02, p = 0.147
Pmean (W·kg^−1^)	8.8±0.4^B,C^	8.3±0.5^A^	8.3±0.6^A^	F2,39 = 4.34, p = 0.020
SAR (cm)	21.8±8.7	21.2±10.5	24.4±9.2	F2,41 = 0.42, p = 0.658
HST (kg·kg^−1^)	1.3±0.2	1.3±0.2	1.4±0.2	F2,41 = 0.70, p = 0.504
SJ (cm)	36.5±4.5^C^	33.5±4.7	31.0±4.8^A^	F2,38 = 4.70, p = 0.015
CMJ (cm)	37.7±3.7^C^	36.4±5.7	32.2±6.2^A^	F2,38 = 3.89, p = 0.029
CMJarm (cm)	46.6±4.1^C^	43.1±6.1	40.6±6.1^A^	F2,38 = 4.05, p = 0.026
Bosco (W·kg^−1^)	38.8±3.7^B,C^	31.0±4.6^A^	33.0±5.5^A^	F2,38 = 10.57, p = 0.000

Data are mean±SD. PWC170 physical working capacity in heart rate 170 beats/min, Pmax=maximal power output estimated by the Force-velocity test, Pmean= mean power during the Wingate anaerobic test, SAR=sit-and-reach test, HST=handgrip strength test, SJ=squat jump, CMJ= countermovement jump, CMJarm countermovement jump with arm-swing.

The letters A, B or C when presenting as exponents denotes that the column’s group is significantly higher compared to the respective group

**Table 3:** Summary of stepwise discriminant analysis by team

		Wilks’ lambda				F			
Step	Entered	Statistic	df1	df2	df3	Statistic	df1	df2	Significance
1	Bosco mean power (W·kg^−1^)	0.599	1	2	34	11.358	2	34	<0.001
2	Stature (cm)	0.454	2	2	34	7.979	4	66	<0.001
